# Mental health stigma: a conundrum for healthcare practitioners in conservative communities

**DOI:** 10.3389/fpubh.2024.1384521

**Published:** 2024-05-15

**Authors:** Wendy A. Booth, Mabrouka Abuhmida, Felix Anyanwu

**Affiliations:** University of South Wales, Treforest, United Kingdom

**Keywords:** mental health, conservative communities, artificial intelligence—AI, Islamic psychology, stigma and shame

## Abstract

This paper presents perspectives on the stigma and shame around mental health in conservative communities, and some of the issues faced by health systems in those communities. The various causes of stigma are explored, and how these are often more pronounced in culturally reserved, conservative communities. While health systems are supposed to provide support for mental health sufferers, this stigma sometimes even extends to healthcare workers, which can discourage patients from asking for assistance. Solutions and reforms are needed, for example education programs; addressing gender norms, and the consideration of culture and religion, to form effective solutions. It is also suggested that alternative therapies and support mechanisms, including digital solutions such as artificial intelligence chatbots, may be useful to provide much needed support to individuals with poor mental health. Along with integrating options such as CBT (cognitive behavioral therapy), it may be useful to draw on indigenous psychologies, such as Islamic psychology, as a way of decolonizing approaches. Therefore, when considering solutions, cultural and religious norms must be considered to ensure their efficacy and acceptance.

## Introduction

This paper presents perspectives on the stigma around mental health in conservative communities, and the association of mental health with shame that is being dealt with by many health systems around the world. This is particularly apparent within developing countries and areas where cultural values and practices may influence treatment options. For example, Elshamy et al. (1) conducted a systematic review of 16 qualitative studies into mental health seeking behaviors in Middle Eastern countries (typically conservative communities), and they discovered negative attitudes toward mental health, including stigma and shame. Furthermore, in a cross-cultural comparison of mental illness stigma and help-seeking attitudes conducted across 16 Arab countries, Fekih-Romdhane et al. (2) found that out of 10,036 respondents, one in four held stigmatizing attitudes toward mental health and negative attitudes toward seeking support.

Encouraging access to mental health support within such conservative communities, where religion, moral and social identity, and the role of the family take precedence, is often difficult (3). This is in part because mental health problems are sometimes viewed as shameful and are, therefore, hidden from friends, family and the workplace (4). Addressing the stigma around mental health is essential to public health provision because it can lead to delays in seeking support, which may exacerbate the condition. In addition, it can have negative consequences not only for sufferers, but also for their families and caregivers, along with poor treatment outcomes due to fears over sharing information about symptoms (5).

A deep understanding of conservative communities and the persistence of the stigma around mental health is essential to ensuring the wellbeing of individuals and the functioning of society as a whole. Therefore, this review of mental health stigma in conservative communities, and the problems faced by the public and healthcare practitioners, adds to the knowledge in this area, and it may be useful in informing future research pathways and findings solutions. Furthermore, while education and “changing the cultural narrative around mental health” [(6), p. 1920] is essential, novel approaches are also needed to directly mitigate the stigma related to mental health conditions. As we enter into the fourth industrial revolution, new digital options and technologies such as artificial intelligence may provide unique solutions.

## Culturally based stigma and shame

The definition of stigma proposed by Goffman (7) posits that stigma is an attribute that devalues a person and sets them aside from others. This definition lays bare the vulnerability of individuals with a disability, especially those with mental health needs. Furthermore, it is well documented in the literature that people with a mental illness (PWMI) face stigma from multiple sources, including schools, hospitals, places of worship and sometimes even from their own families (8, 9). For example, Zolezzi et al. (10) found that the stigma related to mental health in Arab collectivist societies extends beyond the sufferer to the whole family, impacting their reputation; therefore, the stigma and shame around mental health is exacerbated for PWMI due to concerns about the impact on their wider family as well as themselves. Similarly, Andrade et al. (11) explain that in conservative communities where traditional family values and the notion of “honor” exist, “mental health issues are more likely to be perceived as threatening the integrity of the family, given that mental health stigma affects the reputation of the family involved” [(11), p. 2].

The reasons for these negative attitudes toward mental health have been shown to have multiple origins that include culture and tradition, prejudice, and ignorance (12). Although this pattern of behavior is common in many societies, it is often more pronounced among culturally reserved, conservative communities (13), where traditional practices and doctrines have shaped how healthcare workers (HCWs) receive and relate to their clients. Similarly, it has become common place in the literature for authors to draw a link between level of education, mental health literacy and mental health stigma and shame (10, 12, 14). Based on these associations, it has been suggested that mental health stigma is less problematic in populations with higher levels of education or among societies that have high levels of mental health literacy (15). Therefore, it is plausible that knowledge about mental health may provide insights into how individuals perceive and manage mental illness, as well as how the general public interacts with those who suffer from it (16). Despite this, in reality, positive attitudes around PWMI do not always correlate with higher levels of education (17, 18).

## Healthcare systems

Mental health services across conservative communities in the Gulf region have been receiving increasing support, for example, in Qatar, the launch of the National Mental Health Strategy for Qatar in 2013 led to mental health services transitioning toward more community based care within a more comprehensive and integrated system (19). It is notable that across the Gulf region during the COVID-19 pandemic, technology was increasingly used to support mental health sufferers, such as cloud based big data systems, artificial intelligence and AI chatbots, online health communities, and telehealth platforms, were introduced as a response to the pandemic, paving the way for digital mental health solutions. Therefore, Chew et al. (20) (p. 2) claim that “digital mental health tools are the silver lining we are fortunate to have, as they can empower responses to the COVID-19 outbreak at a scale that was never before possible in human history.”

As expected, the health system is supposed to be a haven for people with mental healthcare needs, but structurally, the gaps in the system allow PWMI to fall through the cracks, and for various reasons, HCWs have been implicated in barriers to access for many PWMI through their negative attitudes (21). Furthermore, Knaak et al. (22) explain that stigmatizing attitudes toward mental health suffers are consistent across the stages of healthcare delivery and provision. This is a conundrum that requires further analysis, because in consideration of academic scholarship, and in relation to the general population, HCWs should set a positive example in matters relating to mental health. Indeed, we must consider the opposite argument where there is strong support for HCWs in their capacity as role models and positive change agents and advocates for PWMI (23). However, due to the key role of HCWs in ensuring the best possible care for those who need it, even a singular negative report in this regard is one too many.

Public stigma is believed to be pervasive and holds the potential to foster negative mind-sets that build long-lasting views that may influence important decisions regarding help seeking behaviors (24–26). However, not only does the stigma around mental health manifest itself in the general population, and therefore, the friends and relatives of sufferers, but HCWs are also implicated in the stigma and shaming of PWMI. This connection is especially significant because these individuals act as gatekeepers to healthcare access, therefore PWMI can face an implicit obstacle to accessing the care they need. Moreover, a health workforce lacking in knowledge or with negatively inclined practitioners will lead to the creation of an environment that is unaccommodating for service users. As such, the resulting unfavorable experiences may discourage the care recipient from asking for assistance (27).

## The context of conservative communities

One may argue that there is a contextual basis for the experiences around mental health, which may be due to variations in culture and religion, or purely based on individual differences (22). For instance, there are those who fervently believe that religious convictions can solidify attitudes that shape or form views expressed by individuals (24, 28). In conservative communities, such as some across the Middle East and North Africa (MENA) region, mental health stigma is a prominent topic of concern. This is due in part to its influence on access to mental healthcare, as well as its impact on medical practices in general. Saudi Arabia may be considered as a prime example of one of these communities.

Situated in the MENA region, Saudi Arabia is predominantly an Islamic country, and as such, the views and attitudes toward PWMI and the use of mental healthcare services may have been shaped by cultural practices and religion (6). Accordingly, a Saudi hospital-based study suggests that there may be a bias toward PWMI (29). This can be seen in reports that show that about half of a sample of specialist doctors held stigmatizing views toward PWMI (30). This again shows that individuals with higher education, even among those who are medically trained, may be involved in the stigmatizing and shaming of people with mental healthcare needs (10, 29). Regarding this point, Alamri (29) highlights that in the general population, there is skepticism about taking part in mental health conversations or accessing mental health screening programs. This is based on beliefs that such programs are meant only for people suffering from insanity. In addition, patriarchy is well engrained in many of these communities (31), and as documented, men in these settings are more likely than women to express negative attitudes toward PWMI, even among HCWs (32). This may influence health seeking decisions, particularly for women and their children when they come in contact with a male HCW who may exercise undue control over a patient-doctor encounter. This again may have implications for stigma and shame (31). Accordingly, Awad et al. (4) suggest that psychology solutions should consider religiosity.

Could it be based on these beliefs and practices that some HCWs transfer deeply held views about mental illness in their dealings with those who seek mental health services? Or could it be based on ignorance or poor mental health education among HCW? In conservative communities such as Saudi Arabia, multiple factors may apply, including the diverse background of HCWs, inadequacies of training and, indeed, the influence of culture (32, 33). However, the bottom line is the overall impact of this on limiting access to mental healthcare and the potential to drive PWMI toward the patronage of substandard care (10, 34).

## Discussion

Whatever the root cause of the problem, action to change the attitudes of the general public toward mental health in conservative communities, alongside health reforms and training for HCW, is urgently required. This should include addressing gender norms and the consideration of culture and religion to form effective solutions. According to Alattar et al. (6) (p. 192), to change the cultural narrative around mental illness, “Education and anti-stigma campaigns need to be developed and further training needs to be developed for mental health professionals who need to offer greater support in this area.” In the meantime, alternative therapies and support mechanisms may be useful to provide much needed support to individuals with poor mental health.

With regard to the future of mental health, including in conservative communities, Van Daele et al. (35) state there is a demand for digital therapy that uses psychological theories and methods to interface technology with healthcare to support mental wellness. Furthermore, some have suggested that technology could serve as a bridge between PWMI and healthcare staff or therapists by, as the case may be, offering relief and comfort (35). Cognitive behavioral therapy, or CBT, is a treatment that aims to help people modify their thought processes and behaviors in order to improve their mental health, and its approaches have been integrated into chatbots for mental health. Furthermore, Haque (31) suggests embedding indigenous psychologies to decolonize approaches and ensure culturally appropriate treatment, including Islamic psychology, which considers the nature of the human soul, the development of personality, and the evil eye. This should lead to more rounded solutions and support that is accepted by the communities in which it is offered.

It has been shown that conservative communities face a unique set of problems related to the stigma around mental health and its persistence. While educating healthcare professionals and the public is crucial to changing the cultural narrative, there may also be other alternative approaches that can provide solutions, especially considering the current speed of advance in digital technologies. It may also be possible for religious and cultural norms to be embedded within that technology. That is, given the multiple challenges plaguing the healthcare system in conservative communities, the integration of technology may prove beneficial in enhancing mental health services, especially for individuals suffering from anxiety and depression (36). Although there is a dearth of research on chatbots for mental health in the MENA region, Ujiro et al. (37) report that there may be some progress, as shown in the use of a chatbot called OlloBot, which has been used by doctors to help patients manage their diseases at home and triage patients (37). With this evidence, it becomes imperative to source alternative remedies, such as artificial intelligence tools that are less stigmatizing to PWMI, while attempts are made to improve attitudes and health systems reform and re-strategize to position mental healthcare at the heart of the people they serve.

## Conclusion

It is clear that stigma and shame are a major stumbling block to accessing mental health services in conservative communities. Unfortunately, the stigma extends beyond the individual to their family members, exacerbating the shame they feel. In addition, the healthcare workers who are expected to support those with mental health difficulties also sometimes contribute toward the stigma and feelings of shame, leading to further discouraging patients from seeking assistance. This situation requires a range of responses to address the problem within an environment where mental health problems are on the rise. Such responses should be culturally relevant, religiously appropriate, informative and educational, and utilize cutting edge technologies.

## Author contributions

WB: Writing – original draft, Writing – review & editing. FA: Writing – original draft, Writing – review & editing. MA: Writing – original draft, Writing – review & editing.
